# Genetics of age-at-onset in major depression

**DOI:** 10.1038/s41398-022-01888-z

**Published:** 2022-03-26

**Authors:** Arvid Harder, Thuy-Dung Nguyen, Joëlle A. Pasman, Miriam A. Mosing, Sara Hägg, Yi Lu

**Affiliations:** 1 Department of Medical Epidemiology and Biostatistics, Karolinska Institutet, Stockholm, Sweden; 2 Department of Global Public Health, Karolinska Institutet, Stockholm, Sweden; 3 Department of Cognitive Neuropsychology, Max Planck Institute for Empirical Aesthetics, Frankfurt am Main, Germany; 4 Melbourne School of Psychological Sciences, Faculty for Medicine, Dentistry and Health Sciences, University of Melbourne, Melbourne, Australia

**Keywords:** Clinical genetics, Genomics

## Abstract

Major depression (MD) is a complex, heterogeneous neuropsychiatric disorder. An early age at onset of major depression (AAO-MD) has been associated with more severe illness, psychosis, and suicidality. However, not much is known about what contributes to individual variation in this important clinical characteristic. This study sought to investigate the genetic components underlying AAO-MD. To investigate the genetics of AAO-MD, we conducted a genome-wide association meta-analysis of AAO-MD based on self-reported age of symptoms onset and self-reported age at first diagnosis from the UK Biobank cohort (total *N* = 94,154). We examined the genetic relationship between AAO-MD and five other psychiatric disorders. Polygenic risk scores were derived to examine their association with five psychiatric outcomes and AAO-MD in independent sub-samples. We found a small but significant SNP-heritability (~6%) for the AAO-MD phenotype. No SNP or gene reached SNP or gene-level significance. We found evidence that AAO-MD has genetic overlap with MD risk ($r_g$ = −0.49). Similarly, we found shared genetic risks between AAO-MD and autism-spectrum disorder, schizophrenia, bipolar disorder, and anorexia nervosa ($r_g$ range: −0.3 to −0.5). Polygenic risk scores for AAO-MD were associated with MD, schizophrenia, and bipolar disorder, and AAO-MD was in turn associated with polygenic risk scores derived from these disorders. Overall, our results indicate that AAO-MD is heritable, and there is an inverse genetic relationship between AAO-MD and both major depression and other psychiatric disorders, meaning that SNPs associated with earlier age at onset tend to increase the risk for psychiatric disorders. These findings suggest that the genetics of AAO-MD contribute to the shared genetic architecture observed between psychiatric disorders.

## Introduction

Major depression (MD) is characterized by a pervasive low mood and/or a loss of interest in things normally found enjoyable. Approximately one in six individuals will develop MD during their lifetime [1]. MD is moderately heritable (30–50%) [2], and the largest published genome-wide association study (GWAS) to date has identified 178 independent loci associated with MD risk and estimated the heritability attributable to common variants on the liability scale at 11.3% [3].

MD onsets at all ages, with an average age at onset (AAO-MD) of 30 years [4]. The AAO-MD phenotype is primarily used to dissect MD heterogeneity [5], i.e., using AAO-MD to delineate between cases of early onset and late-onset MD using predetermined age cutoffs. The age cutoffs varied widely between studies [6–9]; some used “absolute” cutoffs, for example considering those that had onset before age 18 as early onset MD [9], while others defined cutoffs with respect to the specific AAO-MD distributions within study samples [6]. In epidemiological studies, early onset MD has been found in association with more severe long-term outcomes, later in life development of bipolar disorder and psychosis, and a greater familial risk [7, 10, 11]. In twin studies, early onset MD was associated with risk for MD in the co-twin, while late-onset MD was associated with vascular damage in the co-twin [8]. Using GWAS data, studies have shown that early onset cases had higher polygenic risk scores (PRS) of MD and are more genetically similar to bipolar disorder and schizophrenia compared to late-onset cases [6, 12].

Although AAO-MD has been implicated as a key clinical characteristic for MD, little is known about what contributes to individual variation in AAO-MD. Studies have examined the role of a genetic component in AAO-MD. Early twin studies suggested twin resemblance in AAO-MD is largely environmental [13]. Recent GWAS showed conflicting results. Two studies estimated that about 17–55% of variances in AAO-MD could be attributable to common variants albeit with large confidence intervals [6, 14], whereas data from the CONVERGE consortium showed that AAO-MD was not significantly heritable [15]. These discrepancies might reflect the methodological challenges in AAO-MD phenotyping, which include measurement differences, sample ascertainment, recall bias, and data censoring. Given the clinical importance of AAO-MD, more research is required to address these methodological challenges.

Here we used the UK biobank (UKB) cohort—the largest genotyped sample to date with comprehensive phenotyping—to study the genetics of AAO-MD. To maximize sample size, we studied two quantitative definitions relevant to AAO-MD: (1) self-reported age of symptoms onset (age at symptom, *N* = 76,365) and (2) self-reported age at first diagnosis (age at diagnosis, *N* = 26,425; or 17,789 after removing overlapping and related samples with the sample subset of age at symptom). Our primary goal was to investigate the genetic architecture of AAO-MD. In addition, we examined the association between PRS of AAO-MD and risk for psychiatric disorders, and vice versa; the association between PRS of psychiatric disorders and AAO-MD.

## Material and methods

### Study population

We used the UKB cohort for all analyses to limit the impact of inconsistent AAO-MD phenotypes across cohorts [6]. The UKB contains deep phenotype and genotype data on ~500,000 individuals aged 37–73 at the date of recruitment and from across the UK. Participants attended a baseline assessment, where information was gathered through digital questionnaires and nurse interviews. A subset of individuals (*N* = 172,751) completed an extended touchscreen questionnaire about depressive and manic symptoms at baseline [16]. In 2017, participants with a valid email address were invited to participate in the online “Mental Health Follow-up” questionnaire (MHQ), where 157,366 individuals completed the questionnaire. For information regarding lifetime depressive symptoms, the International Diagnostic Interview Short Form (CIDI-SF) was used. For nearly the entire population, electronical medical records from hospital care have been further integrated and connected to the UKB data [17]. Informed consent was obtained from all participants. This study used the UK Biobank Resource under Application Number 22224.

### Measures

#### Major depression

**Fig. 1:**
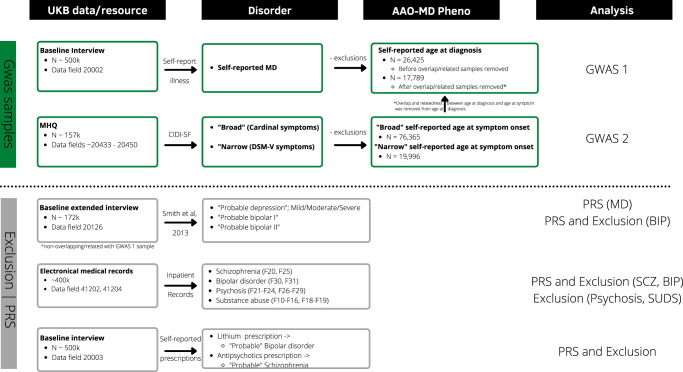
Phenotypic definitions in UK Biobank. This figure displays the data sources used for each definition, and for what purpose the definition was derived. The two MD definitions are marked with green box color. Data sources and definitions for the exclusion criteria, self-reported lithium/antipsychotics prescriptions, probable depression, and probable bipolar disorder type I and II are marked with gray box color.

The comprehensive measures available at the UKB allow for multiple ways to define MD and to thoroughly compare the genetic profiles underlying different definitions [17, 18]. Here we defined MD in two ways: (a) self-reported MD at the baseline interview; and (b) self-reported lifetime symptoms from the CIDI-SF questionnaire in the MHQ (Fig. 1). At the baseline interview, participants were asked to indicate if they (had previously) suffered from any type of illness, including depression. From the MHQ subsample we derived a “broad definition” of MD based on the cardinal symptoms (low mood and anhedonia), and a “narrow definition”, requiring endorsement of either or both of the cardinal symptoms and a minimum of five symptoms (cardinal symptoms included), referred to as the “CIDI” definition in Cai et al. [18] (Supplementary Methods). These MD measures were selected because corresponding age at onset information was available.

Given the phenotypic differences in the two MD definitions, we analyzed them separately and excluded overlapping and related individuals from the subset with self-reported diagnosis. We also excluded individuals with probable schizophrenia, psychosis, and bipolar disorder (for definitions, see *Other psychiatric disorders*, Fig. 1*and* Supplementary Table 1). In addition to this, we applied further exclusion criteria in the “narrow” definition of MD, excluding substance abuse cases, to match the CIDI definition in Cai et al. [18] (Supplementary Methods*)*.

#### Age at onset in major depression

Participants that reported MD during the baseline interview were asked at what age they first received a *diagnosis* of MD from a doctor (data field 20009). We therefore defined *age at diagnosis* for this definition of MD. For participants in the MHQ, if they endorsed either of the cardinal symptoms of MD, they were asked at what age these symptoms first appeared (data field 20433). For this definition of MD, we defined *age at symptom (*Fig. 1*)*.

#### Other psychiatric disorders

We combined the hospital registry data with the self-reported medication data to define cases of probable schizophrenia (F20 or F25 or antipsychotics prescription) and probable bipolar disorder (F30 or F31 or lithium prescription). In addition to this, we extracted cases of the “probable depression” definition described in Howard et al. [19], and cases of probable bipolar type I and II described in Smith et al. [16]. These definitions were used to examine the association between PRS for AAO-MD and risk for these disorders (Supplementary Methods*;* Supplementary Tables 2–4*)*.

### Genotyping, quality control, and ancestry determination

Genotype data were available from the full data release of the UKB in 2018 (*N* = 487,410). UKB samples were genotyped on two customized arrays, the UK Biobank Axiom Array, and the Applied Biosystems UK BiLEVE Axiom Array by Affymetrix covering ~800,000 markers. The genotype data underwent central quality control (QC) and a two-stage imputation process using the reference of the Haplotype Reference Consortium (HRC) and UK10K (for rarer variants not present in HRC). The full QC procedure and pipeline are detailed elsewhere [17]. We applied post-imputation QC to filter imputed variants with minor allele frequency <1% and INFO score <0.6, leaving a total of 9,709,770 variants for analysis. We determined ancestral outliers by projecting the principal components (PCs) of the UKB samples to the PCs of the 1000 Genomes reference panel. We identified 27,700 (5.6%) samples as non-European ancestral outliers (i.e., those with more than three standard deviations from the mean of the European samples in the 1000 Genomes reference) and excluded these individuals from further analysis. The first ten PCs based on the UKB samples were used to adjust for population stratification in subsequent analyses.

### Statistical analysis

#### Phenotype transformation

As there is known confounding between age at onset and age cohort effects for MD [20], we analyzed both definitions with caution. First, since we observed significant correlations between age at symptoms and ${\rm{age}},{\rm{sex}},{\rm{age}}^\ast {\rm{sex}},{\rm{age}}^2$and ${\rm{sex}}^\ast {\rm{age}}^2$, we removed these effects from both phenotypes by analyzing the regression residuals (Supplementary Table 5A, B*)*. Secondly, we transformed the residuals using a rank-based inverse normal transformation.

#### SNP-based heritability

To test how much individual differences in AAO-MD could be explained by common genetic variants we estimated SNP-based heritability $(h_g^2)$ using GCTA-GREML, adjusting for the first ten PCs [21].

#### Genome-wide association study (GWAS) and meta-analysis

We first conducted two AAO-MD GWAS, separately for the age at symptoms and age at diagnosis definitions. Extensive relatedness (over 30% of participants have one or more relatives up to the third degree) was found among UKB participants [17] and removing these samples from analyses would result in a major loss of statistical power. We therefore aimed to account for related samples in GWAS using the fastGWA module in GCTA [22]. We computed a genetic relatedness matrix (GRM) using a filtered set of common SNPs (Supplementary Methods) and thereafter fit a linear-mixed model using the GRM as a mixed effect while adjusting for the first 10 PCs.

In the meta-analysis of the two GWAS results, sample overlap (*N* = 7479) and relatedness (*N* = 1157) were identified and removed before analyses (Supplementary Methods). We subsequently conducted an inverse variance-weighted meta-analysis based on the GWAS summary statistics from the age at symptoms and age of diagnosis definitions using METAL [23]. To examine potential bias from population stratification or relatedness, the intercept was estimated using LD score regression (LDSC) [24].

We used the web-based tool Functional Mapping and Annotation (FuMA) [25], to report and annotate independent genomic loci, and to perform MAGMA gene-based or gene-set analysis and tissue enrichment analysis using GTEx gene expression data [26].

#### Genetic correlations

Using GWAS summary statistics, we examined the genetic correlations (*r*_*g*_) between AAO-MD and major psychiatric disorders to explore the relationship between AAO-MD and genetic risk for these disorders. We obtained publicly available summary statistics for MD [19], schizophrenia [27], bipolar disorder [28], anorexia nervosa [29], autism-spectrum disorder [30], attention-deficit hyperactivity disorder [31], PGC cross-disorder [32] (pooled cases of bipolar disorder, schizophrenia, major depressive disorder, autism-spectrum disorder and attention-deficit hyperactivity disorder) and depressive symptoms [33]. A recent extension of LDSC, the High-Definition Likelihood (HDL) method [34], was used to estimate *r*_*g*_ in all pairs except between AAO-MD and cross-disorder due to a low SNP overlap with the LD-matrix (where we used LDSC instead). Compared to LDSC, HDL improves precision in genetic correlation estimates, as it accounts for the full LD information and it is a likelihood-based method instead of a method of moments. We used the precomputed LD-matrix from UKB individuals which was available with the release of the HDL software. We applied Bonferroni correction to control for multiple testing and consider $r_g$ estimates significant at $p$ < 0.006 (= 0.05/8).

#### Polygenic risk scores (PRSs)

PRSs were derived using the SbayesR module in GCTB [35] (Supplementary Methods) to examine the association between PRS based on meta-analyzed GWAS of AAO-MD (PRS_AAO-MD_). Using PRS_AAO-MD_, we examined its association with risks to mild, moderate, and severe depression, as well as probable bipolar disorder and probable schizophrenia (after removing overlap and restricting to unrelated individuals). Bipolar and psychotic disorders often debut with depressive episodes [36]; we therefore sought to explore whether PRS from these disorders (and from MD) were associated with AAO-MD. PRS for MD (PRS_MD_), schizophrenia (PRS_SCZ_), and bipolar disorder (PRS_BP_) were derived using MD GWAS summary statistics excluding UKB [12] and the largest publicly available summary statistics for schizophrenia and bipolar disorder [27, 28].

To examine the association between PRS_AAO-MD_ with other psychiatric disorders, we fit a logistic regression model. To explore if the potential association was independent of genetic liability related to MD, all models were refit a second time, adjusting for PRS_MD_. Secondly, we estimated the association between PRS_MD,_ PRS_SCZ_, PRS_BP_, and age at symptoms/diagnosis (removing related samples within each definition), to examine the relationship between genetic liability for these disorders and AAO-MD. In all PRS models, we adjusted for the first 10 principal components. To control for multiple testing, we applied a Bonferroni correction and report statistical significance at $p$ < 0.005 (= 0.05/10).

#### Sensitivity analyses

Risk for postpartum depression (PPD) is tied to the reproductive years in women and can complicate the analysis. We therefore, in sensitivity analysis, defined a sample of PPD cases (Supplementary Methods*)* and reran the analysis after removing MD cases that met criteria for PPD.

## Results

### Age at onset of MD is heritable

After QC, 76 365 individuals (64.5% women, 35.5% men) of the 157 366 MHQ participants reported experiencing the cardinal symptoms of MD (age at symptoms *M* = 37.3, SD = 15; Table 1). From the baseline interview, 26 425 individuals after QC (66% women, 34% men) reported previous diagnoses of depression by a doctor (age at diagnosis *M* = 42.7, SD = 13.1; Table 1). Among individuals for whom both measures were available (*N* = 7 479), the correlation between age at symptoms and age at diagnosis was moderate (*r* = 0.54, *p* < 2.2 × 10^−6^).

**Table 1:** Participant characteristics.

	Age at symptoms	Age at diagnosis
*N* analyzed	76,365	26,425
Females, %	64.5%	65.8%
Age, mean (sd)	55.4 (7.67)	55.4 (7.80)
Age at symptoms, mean (sd)	37.3 (15.0)	.^a^
Age at diagnosis, mean (sd)	.^a^	42.7(13.1)
Townsend index, mean (sd)	−1.62 (2.86)	−0.9 (3.25)
Body-mass index (sd)	26.8 (4.76)	28.3 (5.53)
Lifetime-smoking %	44.5%	51.3%

^a^Measure is not available for this definition.

We found evidence for a heritable component underlying the AAO-MD phenotypes, as shown in the significant estimates of $h_g^2$ = 5.6% (95% CI: 4.2–6.9%, *p* < 0.0001) for the age at symptoms (broad MD definition); and for age at diagnosis, $h_g^2$ = 5.7% (95% CI: 2.0–9.2%, *p* = 0.00078). For the narrow MD definition, the $h_g^2$ estimate of the age at symptoms was lower and non-significant ($h_g^2$ = 3.8%, s.e = 4.4%, *p* > 0.05), which could be due to the markedly reduced sample size (19,572, 26% of the sample size in the broad definition).

Despite a lower heritability observed in the narrow MD definition, the genetic correlation between the age at symptoms phenotype in both broad and narrow MD definitions was not significantly different from 1 (*r*_*g*_ = 1.4, s.e = 0.67), indicating a substantial overlap in their underlying genetic factors. In light of this, we proceeded with the age at symptoms phenotype in the broad definition of MD to maximize the sample size in the following analysis.

### No genome-wide significant locus identified in a meta-analysis of nearly 100k individuals

We first conducted GWAS separately within the phenotypes of age at symptoms and age at diagnosis in MD. We observed a strong genetic correlation of 0.91 (s.e = 0.3) between these two phenotypes. Further supporting overlapping genetic components underlying the two AAO-MD phenotypes, we found that PRS of age at symptoms was significantly associated with age at diagnosis (*p* = 2.7 × 10^−5^) *(*Supplementary Table 6*)*.

Given the evidence of a strong genetic overlap between the two phenotypes, we conducted a meta-analysis of AAO-MD combining the GWAS results of the age at symptoms and age at diagnosis together (total *N* = 94 154 after removing overlapping and related samples). No SNP passed the genome-wide significance threshold *(*Supplementary Fig. 1*)*. The intercept from LDSC (1.0051, s.e = 0.0064) indicated negligible confounding from population stratification or relatedness. We did not identify gene-level or gene-set association using MAGMA. However, tissue expression analysis showed significant enrichment in the brain (*p* = 3.2 × 10^−6^), specifically, in the anterior cingulate cortex (*p* = 2.5 × 10^−5^), amygdala (*p* = 3.05 × 10^−5^), frontal cortex (*p* = 5.62 × 10^−5^) and hippocampus (*p* = 1.21 × 10^−5^).

### Shared genetic architecture between age at onset of MD and other psychiatric disorders

**Fig. 2:**
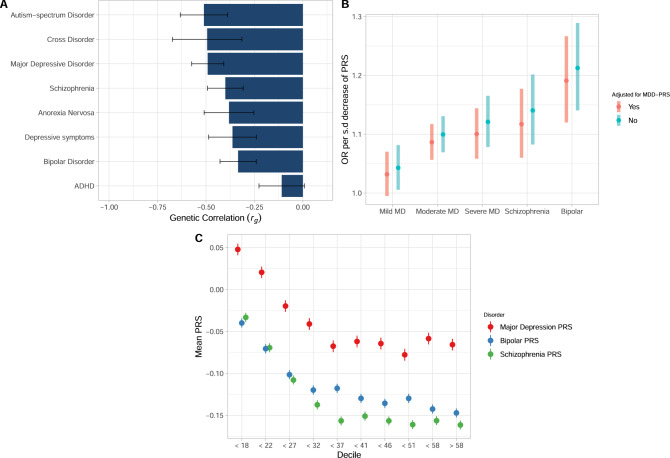
Inverse relationship between AAO-MD and psychiatric disorders. **A** Genetic correlations ($r_g$) estimated using High-Definition Likelihood between summary statistics from the meta-analyzed genome-wide association analysis on age at symptoms/diagnosis of major depression and psychiatric traits. The x-axis displays the $r_g$ estimate with error bars displaying the 95% confidence interval. The y-axis displays the psychiatric trait with which the genetic correlation was calculated. **B** The association between a polygenic risk score derived from genome-wide association analysis from AAO-MD and psychiatric disorders. The y-axis displays the odds ratios of psychiatric disorders per standard deviation decrease in PRS_AAO-MD._ The *x*-axis displays the psychiatric disorder. Error bars display the 95% confidence intervals. The odds ratio estimate is displayed with and without adjustment for MDD-PRS. **C** The mean PRS for major depression, schizophrenia, and bipolar disorder, stratified by age at symptoms decile. The *x*-axis displays each decile for age at symptoms. The y-axis displays the mean PRS for each disorder within a decile. Error bars display 95% confidence interval of the mean PRS in each decile.

Overall, the estimates of $r_g$ were of similar strength between AAO-MD and all included psychiatric disorders except attention-deficit hyperactivity disorder (ADHD) (Fig. 2A). AAO-MD was negatively correlated with psychiatric disorder risks, meaning that SNPs associated with earlier age at onset tend to increase risk for psychiatric disorders. Notably, the genetic correlation of AAO-MD with both MD and depressive symptoms were only of moderate strength ($r_g$ = −0.491, s.e = 0.042 and $r_g$ = −0.363, s.e = 0.062, respectively). The genetic correlation with autism-spectrum disorder ($r_g$ = −0.51, s.e = 0.062), cross-disorders ($r_g$ = −0.494, s.e = 0.091), schizophrenia ($r_g$ = −0.40, s.e = 0.047), bipolar disorder (*r*_*g*_ = −0.334, s.e = 0.047) and anorexia nervosa (*r*_*g*_ = −0.381, s.e = 0.065) were all comparable in magnitude to the correlation between AAO-MD and MD or between AAO-MD and depressive symptoms. ADHD was the only psychiatric disorder that was not significantly related to AAO-MD ($r_g$ = −0.109, s.e = 0.059).

### Polygenic risk scores for AAO-MD are associated with severe psychiatric disorders, independently from MD risk

Using GWAS summary statistics of the meta-analyzed age at diagnosis/symptoms phenotypes, we derived a polygenic risk score (PRS_AAO-MD_) and examined its association with other psychiatric disorders. Overall, the association between PRS_AAO-MD_ and probable depression increased with the severity, with the strongest association with severe MD. PRS_AAO-MD_ association with mild depression was not significant after adjustment for multiple comparisons, but PRS_AAO-MD_ was significantly associated with moderate probable depression (OR = 1.10, *p* = 2.27 × 10^−11^) and severe probable depression (OR = 1.12, *p* = 8.01 × 10^−9^), meaning that for each standard deviation decrease in PRS_AAO-MD,_ the relative risk for moderate and severe depression increased by 11% and 12%. Interestingly, the association was even stronger for probable schizophrenia (OR = 1.14, p = 7.99 × 10^−7^) and probable bipolar disorder (OR = 1.21, *p* = 7.23 × 10^−10^). We further explored if these associations were independent of genetic liability specific to MD by adjusting for PRS_MD_. All associations remained significant (with the exception of mild depression), with slightly attenuated effect sizes (Fig. 2B). We also stratified the analysis by bipolar disorder type I and II and observed similar effect sizes in probable bipolar disorder type I (OR = 1.12, *p* = 0.0119, *N* cases = 512) and probable bipolar type II (OR = 1.15, *p* = 0.00341, *N* cases = 476).

### Polygenic risk scores for psychiatric disorders are associated with age at onset

We found significant associations between PRS_SCZ,_ PRS_BP_, and PRS_MDD_ with age at symptoms and age at diagnosis. Mean PRS_SCZ,_ PRS_BP_, and PRS_MDD_ sharply decrease with age at symptoms among early onset cases (roughly before 32 years old) while stabilizing thereafter; Fig. 2C*)*. In both definitions the strongest effect size was observed for PRS_SCZ_, where the average age at symptoms decreased by approximately one year per standard deviation increase in PRS_SCZ._ The effects of both PRS_MD_ and PRS_BP_ were highly significant; the average age at symptoms decreased by approximately 9 months per standard deviation increase in PRS_BP_, and by approximately 6 months per standard deviation increase in PRS_MD_. In the age at diagnosis subsample, the results were very similar *(*Supplementary Fig. 2*)*.

### Postpartum cases have a negligible impact on the results

Postpartum depression cases tended to onset earlier compared to other MD cases (age at symptoms; mean = 28.1, s.d = 5.99, age at diagnosis; mean = 38, s.d = 12.5, Supplementary Table 7, Supplementary Fig. 3A and B). The correlation between GWAS effects sizes with and without PPD cases was very similar (*r* = 0.98). Removing PPD cases minimally changed the regression coefficients of PRS from other psychiatric disorders (Supplementary Fig. 2).

## Discussion

This study presents the largest GWAS to date on AAO-MD aiming to understand the genetic basis of this important clinical characteristic of MD. We clearly demonstrated that AAO-MD is heritable, with roughly 6% of its variance attributable to common genetic variants. Despite that no single variant or gene passed the genome-wide significance threshold, we had strong evidence for an overall genetic sharing between AAO-MD and MD risk loci (*r*_*g*_ = −0.49), and to a similar extent, between AAO-MD and risks to many other psychiatric disorders (*r*_*g*_ range: −0.3 to −0.5, except for the non-significant *r*_*g*_ with ADHD). Further, we found that PRS_AAO-MD_ was associated with psychiatric disorders (including moderate to severe forms of MD, bipolar disorder and schizophrenia) independently from the genetic liability to MD, and that PRS of these psychiatric disorders were also associated with AAO-MD. Overall, this report provides novel insights into the genetics of AAO-MD, and its relationship with risks to MD and other psychiatric disorders.

Early AAO-MD often correlates with adverse clinical outcomes, including more chronic illness, greater disorder severity, increased suicidality, and lack of treatment response [37, 38]. Therefore, studying AAO-MD may have important clinical implications in primary, secondary, and tertiary prevention. Despite the importance, AAO-MD has been one of the least commonly studied clinical characteristics due to practical difficulties in phenotyping [38]. Here we studied two measurements, age at symptoms and age at diagnosis, using self-reported survey data from UKB. UKB participants reported later AAO-MD (mean age at symptoms = 37.3) than those reported in the literature (mean~22–33 across 18 nations [1], or mean ~29 across PGC-MDD genetic cohorts) [6]. There appeared to be an over five-year delay from the age at symptoms to the age at first diagnosis (mean = 42.7), meaning that many sought treatments at a much later stage than at symptom onset. These observations reveal that on average, UKB participants with MD had later onset. Although this observation could be due to recall bias given the long time gap between the disorder onset and survey participation, it might have partly contributed to the genetic differences observed between UKB cases and other cohorts (*r*_*g*_ = 0.87 with PGC samples) [19].

Despite a moderate phenotypic correlation (*r* = 0.54) between age at symptoms and age at diagnosis, our findings suggest very similar genetic components underlying the two measures. The two measures had nearly identical estimates of SNP-based heritability (5.6% and 5.7%, respectively) and they had a high genetic correlation (0.91), indicating that genetic factors are largely shared. As expected from the high genetic correlation, we showed that PRS of age at symptoms was significantly associated with age at diagnosis. Our significant SNP-based heritability was in line with other reports that showed AAO-MD is heritable; [6, 14] though our estimate based on the large UKB sample was much smaller (~6%; the estimate can be benchmarked against the SNP-heritability of 8.9% [19] for MD risk) than previously estimated, highlighting the need of a large sample size to study this phenotype. Previous research has reported that AAO-MD distribution varied across different cohorts due to major differences in instruments and sample ascertainment [6], making it challenging to study AAO-MD in large numbers. However, our finding of a strong genetic correlation between the two AAO-MD measures suggests that future genetic studies on AAO-MD might benefit from utilizing the large-scale biobanks with electronic medical records to determine age at diagnosis as proxy of AAO-MD.

We have clearly demonstrated an inverse genetic relationship between AAO-MD and risks to psychiatric disorders. Early AAO-MD has been linked to family history of MD, and early AAO-MD in parents confer an increased risk in the offspring compared to the offspring of parents with late AAO-MD [39]. We extended these earlier findings by quantifying the overall genetic sharing between MD risk and AAO-MD, and demonstrating that PRS_AAO-MD_ was associated with MD. We observed a gradient along severity, with a stronger association of PRS_AAO-MD_ in the more severe forms of MD. As early AAO-MD cases tend to have worse long-term outcomes and more severe symptoms [10, 40], our findings revealed that this epidemiological association is partly mediated through genetic liability for AAO-MD. Furthermore, this association was independent of genetic liability to MD, suggesting independent pathways through which AAO-MD genetics confer susceptibility. Interestingly, we found similar levels of genetic overlap between AAO-MD and schizophrenia, bipolar disorder, and that PRS_AAO-MD_ was associated with these two disorders more strongly than with MD. Individuals with psychotic disorders and bipolar disorder frequently experience depressive symptoms before receiving these diagnoses [36], and early onset MD is associated with later bipolar disorder [41]. Hence, our results suggested a genetic basis underlying these clinical observations. For childhood-onset psychiatric disorders, we identified a moderate genetic correlation between AAO-MD and ASD, but not with ADHD. Previous studies showed an association of PRS_SCZ_ with AAO-MD in early onset MD cases, as well as a small association with PRS_BP_ [6, 42]. We replicated these results and further demonstrated that these PRS were also associated with AAO as a quantitative trait, and that it is primarily early onset cases (AAO < 32) that have a higher PRS for these disorders. Altogether, these findings suggest that age at which individuals first experience depressive symptoms may play an important role in psychiatric illness. Clinically, the patient group with early AAO in particular might benefit from closer monitoring and interventions to prevent severe outcomes.

The key strength of this study lies in our use of the large and genetically homogenous sample, with consistent phenotype collection. We also acknowledge several limitations. AAO is a difficult trait to measure and to operat ionalize; self-reported phenotyping suffers from recall bias, while register data miss individuals not seeking treatment and are often left-censored. We carefully analyzed this trait by adjusting for cohort effects and applied transformations on the phenotypes, but it is nonetheless possible that recall bias in the self-reported UKB phenotypes still influenced our results. To define cases of MD, we applied a relatively broad inclusion criteria (the cardinal symptoms of depression or self-reported diagnosis) to maximize sample size, although some argued that minimal phenotyping may bias views of the genetics of MD [18]. Lastly, we acknowledge that UKB has a known healthy volunteer bias, which is further exacerbated in the MHQ subsample [43, 44]. Our results therefore warrant further replication in independent datasets.

Recent advances in statistical methods for case-control GWAS demonstrate that GWAS power can be increased by incorporating age at onset and family history information into case-control GWAS [45]. These methods rely on age at onset being heritable as well as a genetic correlation between age at onset and disease liability to increase statistical power. Therefore, understanding the genetics of age at onset in MD is a necessary first step. In parallel to the ongoing effort in MD genetic discovery, we urge for more genetic studies to investigate key clinical indicators related to MD.

## URLs

FuMa: https://fuma.ctglab.nl/, GCTA: https://cnsgenomics.com/software/gcta/#fastGWA, GCTB: https://cnsgenomics.com/software/gctb/, HDL: https://github.com/zhenin/HDL.

## Supplementary information

Supplementary material

## Data Availability

The code used to generate this analysis is available at https://github.com/Ararder/Genetics-of-age-at-onset-in-MD.
