# Supplementary material for: Genetics of age-at-onset in major depression
Source: Transl Psychiatry. 2022 Mar 26;12:124. doi: 10.1038/s41398-022-01888-z (PMC8960842; doi:10.1038/s41398-022-01888-z)
Supplement: Supplementary file 1 — Supplementary material [file 41398_2022_1888_MOESM1_ESM.docx]

**Supplementary file for**

Genetics of age-at-onset in major depression

Arvid Harder, B.Sc.^1^, Thuy-Dung Nguyen, M.Sc.^1,2^, Joëlle A. Pasman, Ph.D.^1^, Miriam A. Mosing, Ph.D.^1,3,4^, Sara Hägg, Ph.D.^1^, Yi Lu, Ph.D.^1,2^

1. Department of Medical Epidemiology and Biostatistics, Karolinska Institutet, Stockholm, Sweden
2. Department of Global Public Health, Karolinska Institutet, Stockholm, Sweden
3. Department of Cognitive Neuropsychology, Max Planck Institute for Empirical Aesthetics, Frankfurt am Main, Germany.
4. Melbourne School of Psychological Sciences, Faculty for Medicine, Dentistry and Health Sciences, University of Melbourne, Australia.

Table of Contents

[I. SUPPLEMENTARY METHODS 2](#_Toc89335173)

[II. SUPPLEMENTARY TABLES 4](#_Toc89335174)

[Supplementary Table 1: Exclusion criteria for GWAS 4](#_Toc89335175)

[Supplementary Table 2: Psychiatric outcomes for PRS association analysis 4](#_Toc89335176)

[Supplementary Table 3: Definitions of MD 4](#_Toc89335177)

[Supplementary Table 4: List of medications and IDs 5](#_Toc89335178)

[Supplementary Table 5: Cohorts effects for measures of age at onset 6](#_Toc89335179)

[Supplementary Table 5A: Cohort effects for age at symptoms 6](#_Toc89335180)

[Supplementary Table 5B: Cohort effects for age at diagnosis 6](#_Toc89335181)

[Supplementary Table 6: Association of age at diagnosis with age at symptoms PRS 7](#_Toc89335182)

[Supplementary Table 7: Patient characteristics – Postpartum cases 7](#_Toc89335183)

[III. SUPPLEMENTARY FIGURES 8](#_Toc89335184)

[Supplementary Figure 1: Manhattan plot for GWAS of AAO-MD. 8](#_Toc89335185)

[Supplementary Figure 2: Association between PRS from psychiatric disorders with age at symptoms/diagnosis with and without postpartum depression 9](#_Toc89335186)

[Supplementary Figure 3 Distribution of age at symptoms 10](#_Toc89335187)

[**Supplementary Figure 3A**: Histogram of Age at Symptoms 10](#_Toc89335188)

[**Supplementary Figure 3B:** Histogram of Age at Symptoms, postpartum cases only. 10](#_Toc89335189)

[References 11](#_Toc89335190)

1. **SUPPLEMENTARY METHODS**

**Narrow MD (CIDI defined MD)**

The “narrow” definition of MD was derived to as closely as possible mimic the “strictly defined” MD/CIDI-defined MD described in Cai et al (2020)^1^. Cases were participants who endorsed one or both cardinal symptoms (anhedonia; data field 20441 and low mood; data field 20446). In addition to this, cases had to endorse a total of five or more (cardinal symptoms included) symptoms, out of eight possible. In addition to this, participants had to report a significant impact of the depressive episode on their daily lives (data field 20440, encoding “A lot”). Lastly, an additional exclusion criterion of substance abuse disorders was applied using hospital registry data, excluding participants with substance abuse (F10-F14, F16-F19).

**Exclusion criteria**

To restrict our analysis to depression cases, we sought to exclude cases of co-morbid schizophrenia, bipolar disorder and psychosis, using hospital registry data, self-reported medication prescriptions and mental health symptoms from the extended baseline interview (Table 1).

**Probable depression**

For the subset of individuals that participated in the extended baseline interview, there is touchscreen data available regarding mental health symptoms which has been used to derive cases of probable mild, moderate and severe depression (referred to as “probable depression” in Howard et al (2018)^2^. This definition lacks age at onset data and could therefore not be used in our GWAS of age at onset. We extracted the probable mild, moderate and severe cases of depression, and selected cases which were not related to other participants in the UKB, using the kinship variable derived during UKB central QC^3^, and to cases that were non-overlapping with our meta-analysis of AAO-MD.

**Probable bipolar disorder type I and II**

In addition to deriving cases of probable depression, Smith et al (2013)^4^ also derived cases of probable bipolar disorder type I or II using the additional data from the extended touchscreen questionnaire. This definition was used both as an exclusion criterion, and as a phenotype to predict using PRS from AAO-MD.

**Probable cases of schizophrenia and bipolar disorder**

We combined the ICD-coded schizophrenia and bipolar disorder cases and combined them with the self-reported medication data to define cases of probable schizophrenia (F20 or F25 or self-reported antipsychotics prescription) and probable bipolar disorder (F30 or F31 or self-reported lithium prescription). We included cases which were not related to other participants and removed overlap with the meta-analysis sample.

**Postpartum depression**

Postpartum depression (PPD) cases were identified from three sources: women who reported post-natal depression during the nurse interview at the baseline recruitment (UKB field: 20002, code 1531); cases with MD cardinal symptoms related to childbirth (UKB field: 20445); and women with ICD diagnosis of mental and behavioral disorders associated with the puerperium (F53 in UKB fields: 41202, 41204).

**Controls**

As controls in the PRS association analysis, we aimed to define a set of participants without any indication of MD, and therefore used several MD definitions (Table 3) to filter away possible MD cases. Remaining participants were filtered to retain a sample of unrelated individuals of European descent as controls.

**Genome-wide association studies (GWAS)**

GWAS were conducted separately within the age at diagnosis and the age at symptoms samples. Using fastGWA we were able to retain related individuals within each sample. However, fastGWA could not adjust for the relatedness *between* the two definitions. We therefore estimated relatedness between the two definitions by deriving third-degree kinships or higher among all non-overlapping individuals using KING^5^, and in each kinship pair removed the individual in the age at diagnosis definition.

FastGWA requires a genetic relatedness matrix (GRM) to quantify and adjust for relatedness between individuals in the GWAS sample. We estimated the GRM for the ~459,000 individuals that passed our quality control and filtering. To reduce computational requirements we reduced the set of SNPs by following a similar procedure as in the original publication,^6^ by filtering down to a set of HapMap3 SNPs without the MHC region and MAF > 0.1. We then lightly pruned the set of SNPs in Plink 1.9 using the indep-pairwise command (window size = 1000, stepsize = 100, r^2^ = 0.9). This resulted in 575,805 SNPs. The computation was divided into 300 parts using the –grm-parts command, with each job using one core with 7GB memory. The sparse GRM was derived using the –grm-sparse flag, setting all elements below 0.05 to 0.

**Polygenic risk scores**

The polygenic scores were derived using the –bayes R command in GCTB (<https://cnsgenomics.com/software/gctb/#Overview>). In rescaling GWAS summary statistics, we used the parameters specified in the original publication^7^; --pi 0.95, 0.2, 0.2, 0.1, --gamma 0 , 0.1, 0.1, 1, --exclude-mhc, --chain-length 10,000, --burn-in 4000. Polygenic risk scores based on rescaled summary statistics was then computed using plink2 with the –score and –variance-standardize commands. Prior to deriving PRS, the set of SNPs from the summary statistics were restricted to high quality SNPs with INFO > 0.9 and MAF > 0.1, as these SNPs have been shown to generate the most robust results^8^.

1. **SUPPLEMENTARY TABLES**

## **Supplementary Table 1**: Exclusion criteria for GWAS

| **Disorder** | **Source** | **Data fields** | **Encoding** | **N^1^** |
| --- | --- | --- | --- | --- |
| Schizophrenia | Hospital admission records | 41202, 41204 | F20, F25 | 884 |
| Bipolar Disorder | Hospital admission records | 41202, 41204 | F30, F31 | 1364 |
| Psychosis | Hospital admission records | 41202, 41204 | F21-F24, F26-F29 | 608 |
| Antipsychotics | Self-reported medication data | 20003 | Table 4 | 2330 |
| Lithium | Self-reported medication data | 20003 | Table 4 | 824 |
| Probable Bipolar 1 | Extended baseline interview | 20126 | 1 | 807 |
| Probable Bipolar 2 | Extended baseline interview | 20126 | 2 | 808 |

^1^Sample sizes are before quality control and filters

## **Supplementary Table 2**: Psychiatric outcomes for PRS association analysis

| **Disorder** | **Source** | **Data fields** | **Encoding** | **N^1^** | |
| --- | --- | --- | --- | --- | --- |
| Probable Schizophrenia | Hospital admission records,  Self-reported medication | 41202, 41204,  20003 | F20, F25, Antipsychotics (Table 4) | 1553 | |
| Probable Bipolar Disorder | Hospital admission records,  Self-reported medication | 41202, 41204,  20003 | F30, F31,  Lithium (Table 4) | 1123 | |
| Mild MD | Extended baseline interview, | 20126 | 5 | 3219 | |
| Moderate MD | Extended baseline interview, | 20126 | 4 | 5596 | |
| Severe MD | Extended baseline interview, | 20126 | 3 | 2836 | |
| Probable Bipolar 2 | Extended baseline interview | 20126 | 2 | 476 | |
| Probable Bipolar 1 | Extended baseline interview | 20126 | 1 | 512 | |
| Controls | Hospital admission records, Self-reported medication,  Baseline interview,  Extended baseline interview. | - | Table 3 | 171,224 | |
| ^1^Sample sizes are after quality control and filters | | | | |  |

## **Supplementary Table 3**: Definitions of MD

| Definition | Data field | Criteria for case |
| --- | --- | --- |
| Cardinal symptoms | Ever had prolonged loss of interest in normal activities (UKB 20441); or prolonged feelings of sadness or depression (UKB 20446) | 20441 = 1 or 20446 = 1 |
| Self-reported MD | Self-reported depression (20002)  Self-reported Mental health problems ever diagnosed by a professional (UKB 20544) | UKB20002 = 1286 or  UKB20544 = 1 |
| ICD-coded MD | Primary or secondary MD diagnosis from linked hospital registry data (41202 and 41204) | 41202 = F32/F33/F34/F38/F30  41204 = F32/F33/F34/F38/F30 |
| Probable MD (Smith et al) | Probable mild/moderate/severe MD  20126 | 20126 = 3 or 4 or 5 |
| Help-seeking MD | Ever seen a psychiatrist for nerves, anxiety, tension or depression. (2010)  Ever seen a GP for nerves, anxiety, tension or depression. (2090) | 2090 = 1 or 2010 = 1 |
| Antidepressant use | Self-reported use of any antidepressant (20003) | Any code in the list of anti-depressants |

## **Supplementary Table 4**: List of medications and IDs

| **ID^a^** | **Medication name** | **ID^a^** | **Medication name** |
| --- | --- | --- | --- |
| **Antidepressant** | | | |
| 1140879616 | amitriptyline | 1141151978 | reboxetine |
| 1140921600 | citalopram | 1141152736 | zispin |
| 1140879540 | fluoxetine | 1140867640 | doxepin |
| 1140867878 | sertraline | 1140867920 | moclobemide |
| 1140916282 | venlafaxine | 1140867850 | phenelzine |
| 1140909806 | dosulepin | 1140879544 | fluvoxamine |
| 1140867888 | paroxetine | 1141200570 | yentreve |
| 1141152732 | mirtazapine | 1140867934 | triptafen |
| 1141180212 | escitalopram | 1140867758 | surmontil |
| 1140879634 | trazodone | 1140867914 | tranylcypromine |
| 1140867876 | prozac | 1140867820 | allegron |
| 1140882236 | seroxat | 1141151982 | edronax |
| 1141190158 | cipralex | 1140882244 | molipaxin |
| 1141200564 | duloxetine | 1140879556 | mianserin |
| 1140867726 | lofepramine | 1140867852 | nardil |
| 1140879620 | clomipramine | 1140867860 | faverin |
| 1140867818 | nortriptyline | 1140917460 | nefazodone |
| 1140879630 | imipramine | 1140867938 | Amitriptyline + chlordiazepoxide |
| 1140879628 | dothiepin | 1140867856 | isocarboxazid |
| 1141151946 | cipramil | 1140867922 | manerix |
| 1140867948 | amitriptyline | 1140910820 | maoi |
| 1140867624 | prothiaden | 1140882312 | sinequan |
| 1140867756 | trimipramine | 1140867944 | Tranylcypromine + trifluoperazine |
| 1140867884 | lustral | 1140867784 | ludiomil |
| 1141201834 | cymbalta | 1140867812 | norval |
| 1140867690 | anafranil | 1140867668 | tryptizol |
| **Antipsychotic** | | | |
| 1140928916 | olanzapine | 1141202024 | abilify |
| 1141152848 | quetiapine | 1140882098 | fluphenazine |
| 1140867444 | risperidone | 1140867184 | haldol |
| 1140879658 | chlorpromazine | 1140867092 | serenace |
| 1140868120 | trifluoperazine | 1140882320 | clozaril |
| 1141153490 | amisulpride | 1140910358 | cpz |
| 1140867304 | sulpiride | 1140867208 | perphenazine |
| 1141152860 | seroquel | 1140909802 | levomepromazine |
| 1140867168 | haloperidol | 1140867134 | pericyazine |
| 1141195974 | aripiprazole | 1140867306 | dolmatil |

| 1140867244 | stelazine | 1140867210 | fentazin |
| --- | --- | --- | --- |
| 1140867152 | depixol | 1140867398 | fluphenazine |
| 1140909800 | flupentixol | 1140867078 | benperidol |
| 1140867420 | clozapine | 1140867218 | pimozide |
| 1140879746 | promazine | 1141201792 | zaponex |
| 1141177762 | risperdal | 1141200458 | denzapine |
| 1140867456 | modecate | 1140867136 | neulactil |
| 1140867952 | fluanxol | 1140879750 | thioridazine |
| 1140867150 | flupenthixol | 1140867180 | dozic |
| 1141167976 | zyprexa | 1140867546 | fluspirilene |
| 1140882100 | zuclopenthixol | 1140928260 | panadeine |
| 1140867342 | clopixol | 1140927956 | sertindole |
| 1140863416 | largactil |  |  |
| **Lithium** | | | |
| 1140867490 | lithium product | 1140867504 | priadel 200mg m/r tablet |
| 1140867494 | camcolit 250 tablet | 1140867518 | litarex 564mg m/r tablet |
| 1140867498 | liskonum 450mg m/r tablet | 1140867520 | li-liquid 5.4mmol/5ml oral solution |
| 1140867500 | phasal 300mg m/r tablet |  |  |
| *This list is* ^9^*\| ^a^ID in UKB data (field 20003)* | | | |

## **Supplementary Table 5**: Cohorts effects for measures of age at onset

### Supplementary Table 5A: Cohort effects for age at symptoms

| Linear model | Beta | SE | T-value | P-value | N | R^2^ | R^2^ adj |
| --- | --- | --- | --- | --- | --- | --- | --- |
| Intercept | -17.13 | 9.37 | -1.82 | 0.067 | - | - | - |
| Age | 1.55 | 0.35 | 4.48 | 7.36e-06 | - | - | - |
| Sex | 20.4255 | 5.55 | 3.71 | 0.000206 | - | - | - |
| Age*sex | -0.74 | 0.2 | -3.66 | 0.000252 | - | - | - |
| Age^2^ | -0.0087 | 0.003 | -2.77 | 0.005568 | - | - | - |
| Age^2^*sex | 0.0058 | 0.0018 | 3.147 | 0.001649 | - | - | - |
| Model | - | - | - | - | 76365 | 0.055 | 0.055 |
| *F-statistic: 900.4 on 5 and 76359 df, p-value < 2.2x10^-16^* | | | | | | | |

### Supplementary Table 5B: Cohort effects for age at diagnosis

| Linear model | Beta | SE | T-value | P-value | N | R^2^ | R^2^ adj |
| --- | --- | --- | --- | --- | --- | --- | --- |
| Intercept | 0.72 | 16.18 | 0.045 | 0.96 | - | - | - |
| Age | 0.91 | 0.59 | 1.54 | 0.124 | - | - | - |
| Sex | -11.86 | 9.4 | -1.21 | 0.207 | - | - | - |
| Age*sex | 0.44 | 0.347 | 1.268 | 0.205 | - | - | - |
| Age^2^ | -0.002 | 0.005 | -0.328 | 0.743 | - | - | - |
| Age^2^*sex | -0.004 | 0.003 | -1.454 | 0.146 | - | - | - |
| Model | - | - | - | - | 17789 | 0.146 | 0.146 |
| *F-statistic: 610.2 on 5 and 17783 df, p-value < 2.2x 10^-16^* | | | | | | | |

##

## **Supplementary Table 6**: Association of age at diagnosis with age at symptoms PRS

| Variables | Beta | SE | T-value | P-value | N | R^2^ | R^2^ adj |  |
| --- | --- | --- | --- | --- | --- | --- | --- | --- |
| AAO_PRS | 0.509 | 0.119 | 4.27 | 5.47x10^-6^ | 12,826 | 0.003 | 0.002 |  |
| F-statistic: 3.185 on 11 and 12814 DF, p-value: 0.0002479 | | | | | | | | |
|  | | | | | | | | |

## **Supplementary Table 7**: Patient characteristics – Postpartum cases

| Variables | **Age at symptoms** | **Age at diagnosis** |
| --- | --- | --- |
| N analysed | 5800 | 849 |
| Age, mean (sd) | 55.5 (7.76) | 54.3 (7.67) |
|  |  |  |
| Age at symptoms, mean (sd) | 28.1 (5.99) |  |
| Age at diagnosis, mean (sd) |  | 38.2 (12.4) |
| Townsend, mean (sd) | -1.93 (2.656) | -1.73 (2.82) |
| BMI | 26.8 (5) | 27.7 (5.43) |
| Lifetime-smoking | 42.5% | 43% |

##

1. **SUPPLEMENTARY FIGURES**


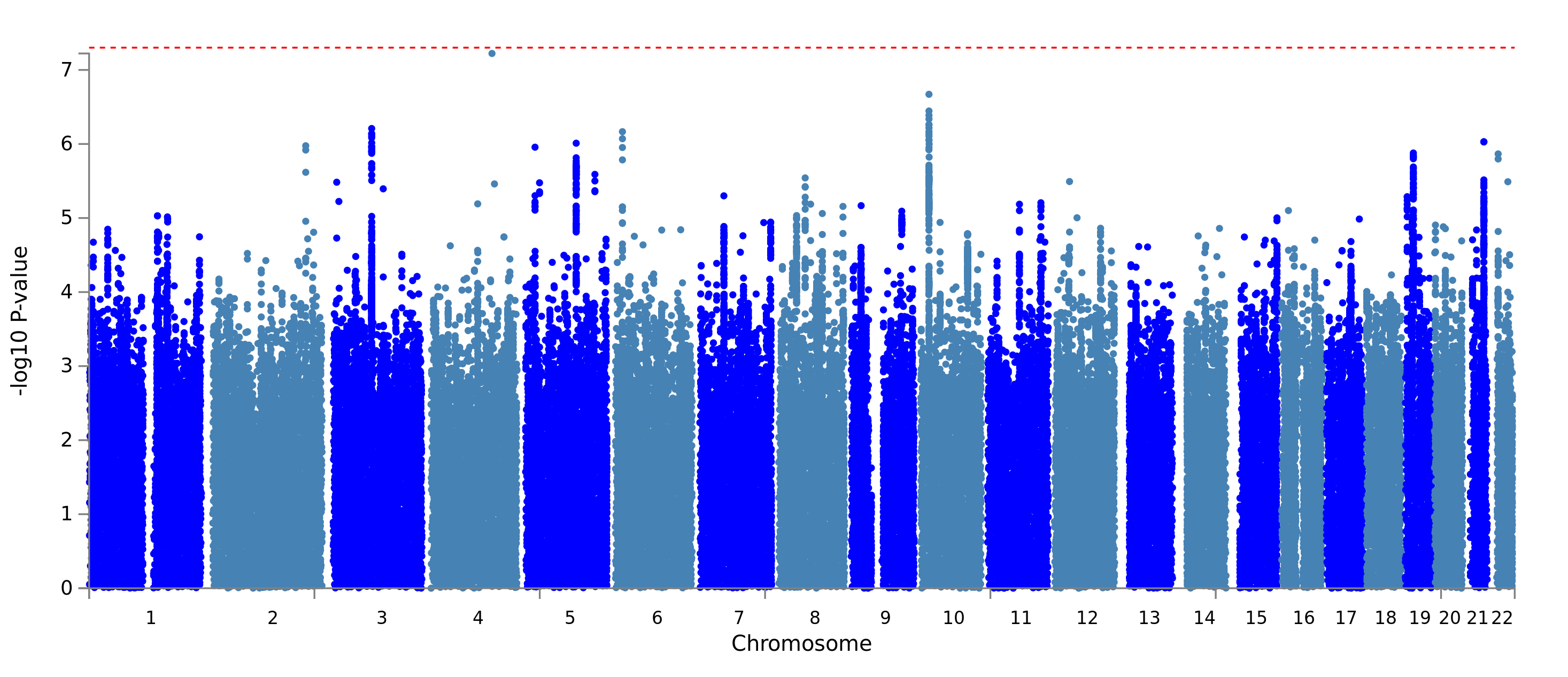


## **Supplementary Figure 1:** Manhattan plot for GWAS of AAO-MD.

Manhattan plot for the meta-analysis of the genome-wide association studies on age at symptoms and age at diagnosis of major depression (combined n = 94,154). The x-axis displays genomic position (and chromosome). The y-axis displays the –log_10_ (*P*) *z* statistic for SNPs.

**
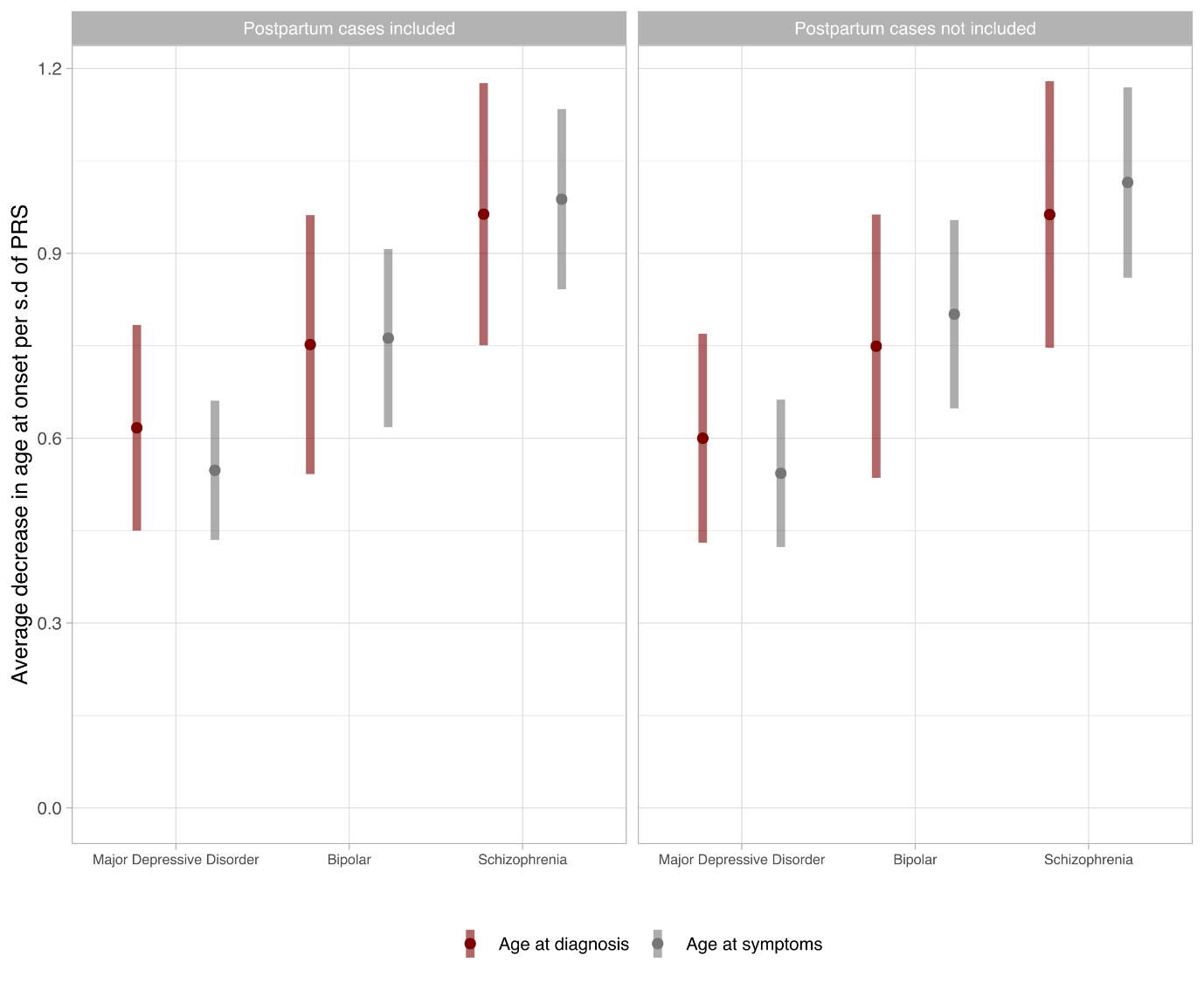
**

## **Supplementary Figure 2**: Association between PRS from psychiatric disorders with age at symptoms/diagnosis with and without postpartum depression

The y-axis displays the regression coefficient (decrease in age at symptoms/diagnosis per s.d decrease in AAO-MD_PRS_, in years) and the x-axis displays from which psychiatric disorder the PRS was derived. Color differentiates between the age at symptoms and age at diagnosis cohort. Error bars mark the 95% confidence interval of the regression coefficient. The left figure displays results with postpartum cases included, the right figure with postpartum cases removed.

## **Supplementary Figure 3:** Distribution of age at symptoms


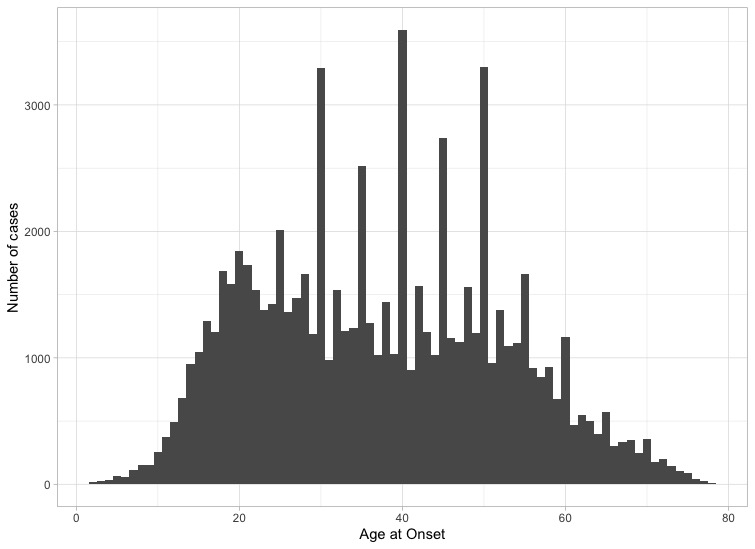


### **Supplementary Figure 3A**: Histogram of Age at Symptoms

The x-axis displays age at symptoms, and the y-axis displays the number of participants that report an onset for each distinct year.


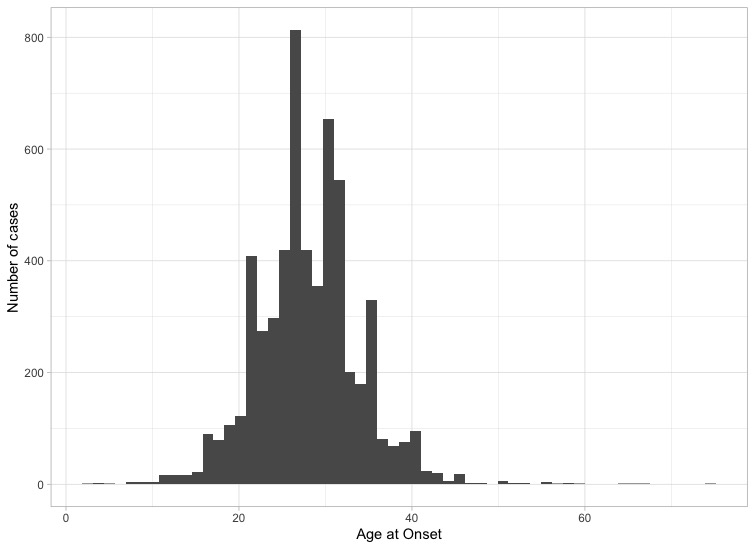


### **Supplementary Figure 3B:** Histogram of Age at Symptoms, postpartum cases only.

The x-axis displays age at symptoms, and the y-axis displays the number of participants that report an onset for each distinct year. The sample was restricted to display data only for participants that meet criteria for postpartum depression.

# References

1 Cai N, Revez JA, Adams MJ, Andlauer TFM, Breen G, Byrne EM *et al.* Minimal phenotyping yields genome-wide association signals of low specificity for major depression. *Nat Genet* 2020; **52**: 437–447.

2 Howard DM, Adams MJ, Clarke T-K, Hafferty JD, Gibson J, Shirali M *et al.* Genome-wide meta-analysis of depression identifies 102 independent variants and highlights the importance of the prefrontal brain regions. *Nat Neurosci* 2019; **22**: 343–352.

3 Bycroft C, Freeman C, Petkova D, Band G, Elliott LT, Sharp K *et al.* The UK Biobank resource with deep phenotyping and genomic data. *Nature* 2018; **562**: 203–209.

4 Smith DJ, Nicholl BI, Cullen B, Martin D, Ul-Haq Z, Evans J *et al.* Prevalence and Characteristics of Probable Major Depression and Bipolar Disorder within UK Biobank: Cross-Sectional Study of 172,751 Participants. *PLoS ONE* 2013; **8**: e75362.

5 Manichaikul A, Mychaleckyj JC, Rich SS, Daly K, Sale M, Chen W-M. Robust relationship inference in genome-wide association studies. *Bioinformatics* 2010; **26**: 2867–2873.

6 Jiang L, Zheng Z, Qi T, Kemper KE, Wray NR, Visscher PM *et al.* A resource-efficient tool for mixed model association analysis of large-scale data. *Nat Genet* 2019; **51**: 1749–1755.

7 Lloyd-Jones LR, Zeng J, Sidorenko J, Yengo L, Moser G, Kemper KE *et al.* Improved polygenic prediction by Bayesian multiple regression on summary statistics. *Nat Commun* 2019; **10**: 5086.

8 Ni G, Zeng J, Revez JA, Wang Y, Zheng Z, Ge T *et al.* A Comparison of Ten Polygenic Score Methods for Psychiatric Disorders Applied Across Multiple Cohorts. *Biol Psychiatry* 2021. doi:10.1016/j.biopsych.2021.04.018.
